# The species origin of the serum in the culture medium influences the *in vitro* toxicity of silica nanoparticles to HepG2 cells

**DOI:** 10.1371/journal.pone.0182906

**Published:** 2017-08-10

**Authors:** Cédric Pisani, Estelle Rascol, Christophe Dorandeu, Jean-Charles Gaillard, Clarence Charnay, Yannick Guari, Joël Chopineau, Jean Armengaud, Jean-Marie Devoisselle, Odette Prat

**Affiliations:** 1 Institut Charles Gerhardt de Montpellier, MACS, UMR 5253 CNRS-ENSCM-UM, Place Eugène Bataillon, Montpellier, France; 2 CEA, Direction de la Recherche Fondamentale—BIAM, Site de Marcoule, Bagnols-sur-Cèze, France; 3 CEA, Direction de la Recherche Fondamentale—IBITECS, Site de Marcoule, Bagnols-sur-Cèze, France; 4 Institut Charles Gerhardt de Montpellier, IMNO, UMR 5253 CNRS-ENSCM-UM, 1701, Place Eugène Bataillon, Montpellier, France; 5 Université de Nîmes Rue Georges Salan, Nîmes, France; Universidad de Castilla-La Mancha, SPAIN

## Abstract

The formation of a protein corona around nanoparticles can influence their toxicity, triggering cellular responses that may be totally different from those elicited by pristine nanoparticles. The main objective of this study was to investigate whether the species origin of the serum proteins forming the corona influences the *in vitro* toxicity assessment of silica nanoparticles. Coronas were preformed around nanoparticles before cell exposures by incubation in fetal bovine (FBS) or human (HS) serum. The compositions of these protein coronas were assessed by nano-LC MS/MS. The effects of these protein-coated nanoparticles on HepG2 cells were monitored using real-time cell impedance technology. The nanoparticle coronas formed in human or fetal bovine serum comprised many homologous proteins. Using human compared with fetal bovine serum, nanoparticle toxicity in HepG2 cells decreased by 4-fold and 1.5-fold, when used at 50 and 10μg/mL, respectively. It is likely that “markers of self” are present in the serum and are recognized by human cell receptors. Preforming a corona with human serum seems to be more appropriate for *in vitro* toxicity testing of potential nanocarriers using human cells. *In vitro* cytotoxicity assays must reflect *in vivo* conditions as closely as possible to provide solid and useful results.

## Introduction

Magnetic mesoporous silica nanoparticles (M-MSNs) are of particular interest in nanomedicine as targeting tools for theranostics: the combined discipline of therapeutics and diagnostics [1–3]. These nanoparticles (NPs) are intended for development as injectable nanocarriers for drug delivery, but their safety must first be established. Indeed, nanomedicine and nanotoxicology are two sides of the same coin, the main difference between the toxicological and pharmacological aspects being whether or not a specific effect is desired [4, 5]. While the toxicity of drug-loaded nanocarriers is wanted against targeted tissues, the biocompatibility of the carrier itself must be investigated to avoid collateral toxicity, especially for the liver which is the main accumulation organ of mesoporous silica nanocarriers [6–8].

Although animal experimentation remains the gold standard in regulatory toxicology, it is being replaced where possible with *in vitro* cytotoxicity assays using human cell cultures, for both ethical considerations and scientific reasons regarding the limits to the information that can be gained by extrapolation from animals to humans [9–11]. *In vitro* cell culture assays provide high-throughput systems for rapid and cost-effective hazard screening. At present, NP cytotoxicity testing is based on the same *in vitro* methods established for the hazard characterization of chemicals. Nevertheless, nanotoxicology is a special area of toxicology, with evidence accumulating that the effects of NPs differ widely from those of chemicals, and that they may interfere with cell cultures and commonly used test systems [12]. This is due to the specific features (particle size, size distribution, density, surface modification, aggregation/agglomeration state, shape) that confer on NPs their special physical properties. According to the REACH Nano Consultation [13], further research is required to gain a better understanding of how representative experimental conditions are of real human exposure conditions, which parameters differ, and how these may affect the observed toxicity. The challenge for NP toxicity testing is the development of new, standardized *in vitro* methods that cannot be affected by the NP properties [12].

In biological fluids or cell media, NPs are known to adsorb a variety of biomolecules, especially proteins, forming a layer called the corona. In particular, in contact with blood, NPs are covered with multiple human serum proteins, which control the NP cellular fate and potential toxicity. The corona around NP creates a new nano-object, whose interactions with living cells are different from those induced by pristine NP [14, 15]. The protein corona is influenced by the physico-chemical parameters of NPs (size, shape, surface charge, atom composition) and proteins (charge, plasticity, conformation) [16, 17], and also by biological parameters (protein-protein interactions, biomolecule composition) [18, 19]. Some experimental protocols in the nanotoxicology field are available in the public domain. For instance, the European Joint Action Nanogenotox proposed an NP dispersion protocol using bovine serum albumin (BSA) solution to avoid NP aggregation in biological media [20]. For *in vitro* cell assays, the Nanotechnology Characterization Laboratory (NCL) provides protocols such as an MTT cytotoxicity assay on HepG2 cells using fetal bovine serum (FBS) in cell culture (NCL Method GTA-2) [21].

In cellular toxicology studies, the supplementation of FBS in cell culture media is a convention that is followed for practical and economic reasons. FBS contains all the factors required for cell growth, stimulation of cell proliferation, and cellular metabolism, and is included in the majority of growth supplements used in the culture of human and animal cells. Nevertheless, in the special case of NPs, the systematic use of animal sera in cell culture for toxicology studies raises concerns about the correct evaluation of cytotoxicity. There is no doubt that the presence of adsorbed proteins influences the biological and toxicological fates of NPs, even if it is difficult to determine how the protein composition of the corona affects cell behavior. According to Anders *et al*., the improved dispersion stability induced by FBS leads to increased NP bioavailability in suspension cell models, and reduced NP sedimentation onto adherent cell layers, resulting in more accurate *in vitro* toxicity assessments [22]. NP uptake has also been shown to be mediated by the protein corona [23, 24].

Despite the advantages of standardized protocols, the impact of the formation of a protein corona on nanomaterial toxicity evaluations needs to be taken into consideration. Although there is scientific interest in the corona, its influence on the NP cytotoxicity remains unclear [25].

In this study, we analyzed the impact of the species origin of the serum on the assessment of nanotoxicity in cell cultures *in vitro*. For this, we built preformed coronas around M-MSNs by incubation in sera of different species before exposing cells to NPs. These nanoparticles intended to be used in nanomedicine are reproducible and well characterized [26]. The protein content of these coronas, composed of either fetal bovine serum (FBS) or human serum (HS) proteins, was firstly identified by mass spectrometry (nano-LC MS/MS). Hepatic HepG2 cells were then exposed to these NPs, with or without a preformed corona. Cellular effects were analyzed by real-time cell impedance technology (xCELLigence System, ACEA Biosciences). This technology, a cell-based microelectronic biosensor which provides real-time and label-free cellular analyses, allows the limits of endpoint analysis to be extended by capturing data throughout the entire time-course of an experiment in order to obtain data that are more physiologically relevant. The xCELLigence platform is a powerful and reliable tool that can be used for toxicity and pharmacology studies [27].

## Materials and methods

### 1. Synthesis of magnetic mesoporous silica nanoparticles (M-MSNs)

Magnetic mesoporous silica nanoparticles (M-MSN) were synthesized according to an optimized procedure, previously described [26]. This two-step method allows the formation of monodisperse and homogeneous core@shell Fe_3_O_4_@MSN nanoparticles with a single magnetic iron oxide core per nanoparticle surrounded by a mesoporous silica shell.

### 2. Nanoparticle characterization

Transmission electron micrographs (TEM) were obtained using a JEOL 1200 EX II microscope.

Hydrodynamic diameters and zeta potentials were determined using a Zetasizer Nano ZS (Malvern Instruments Ltd, UK). Measurements (n = 3) were performed at 20 μg/mL NPs after sonication for 2 min in an ultrasonic bath (Elma Transsonic T780/H) in 1X PBS for hydrodynamic diameters and in 20 mM HEPES, 5 mM NaCl buffer for zeta potentials at 20°C, pH 7.4.

### 3. Formation of a preformed corona

A stock suspension of M-MSNs (1 mg/mL) in 1X PBS (Invitrogen) was prepared. After sonication in an ultrasonic bath (Elma Transsonic T780/H) at 4°C for 2 min, the M-MSN suspension was supplemented with 10% HS (Sigma-Aldrich) or HyClone™ FBS (Thermo Fisher Scientific). Exposure started at the time of serum addition. Twenty-four hours after the addition of serum, samples containing M-MSNs coated with proteins were washed three times with 1 mL 1X PBS (Invitrogen) by gently mixing, followed by magnetization separation for 30 s (1.4 Tesla magnets). Washing steps were necessary to eliminate non-adsorbed proteins.

### 4. Sample preparation for nano-LC mass spectrometry

M-MSNs coated with proteins were suspended in 20 μL 1X PBS and 10 μL 1X LDS (lithium dodecyl sulfate, Invitrogen). 1X LDS working solution contained: 106 mM Tris/HCl, 141 mM Tris base, 2% lithium dodecyl sulfate, 10% glycerol, 0.51 mM EDTA, 0.22 mM G250 SERVA® Blue, 0.175 mM Phenol Red, buffered at pH8.5 and was supplemented with 2.5% beta-mercaptoethanol. Samples were heated at 99°C for 5 min and loaded onto a 4–12% NuPAGE gel (Invitrogen) for a short (5 min) denaturating electrophoresis run at 200 V in 1X MES/SDS running buffer (2-(*N*-morpholino(ethanesulfonic acid)) from Sigma Aldrich. Proteins migrated into the gel, whilst nanoparticles were retained in the wells. Gels were stained with Coomassie Blue Safe stain (Invitrogen). Densitometric analyses of polyacrylamide gels were performed using Quantity One software (Biorad).The polyacrylamide bands containing all proteins were processed as previously described [28]. Briefly, after overnight destaining at 4°C with milliQ water, the protein content from each well was excised with a scalpel as a single polyacrylamide band. These bands were treated with 25 mM DTT, then with iodoacetamide and finally proteolyzed with 0.01% proteasMAX (Promega). The resulting peptides (10 μL) were analyzed using an ESI-Q Exactive HF mass spectrometer (ThermoFisher Scientific) incorporating an ultra-high-field Orbitrap analyzer and coupled to an Ultimate 3000 RSL Nano LC System (Dionex-LC Packings). For nano-liquid chromatography, samples were loaded and desalted on-line on a reverse phase Acclaim PepMap100 C18 micro precolumn (5 μm, 100 Å, 300 μm internal diameter x 5 mm, Thermofisher) and resolved on an Acclaim PepMap100 C18 nano column (3 μm, 100 Å, 75 μm internal diameter x 50 cm, Thermofisher) at a flow rate of 0.2 μL/min, with a 4–25% gradient of solvent B (80% acetonitrile, 20% water, 0.1% formic acid) against solvent A (0.1% formic acid, 99.9% water) for 70 min and then 25–40% for 20 min, for a total gradient run time of 90 min. A top20 data-dependent method was used for MS/MS spectrum acquisition. Full-scan mass spectra were measured from 350 to 1500 m/z with an Automatic Gain Control Target set at 3x10^6^ ions and a resolution of 60,000. MS/MS scan was initiated at a resolution of 15,000 for ions with potential charge of 2+ and 3+ and with a dynamic exclusion of 10 s. MS/MS were recorded with an Automatic Gain Control Target set at 1x10^5^ ions.

### 5. Protein identification and proteomic quantification

MS/MS spectra were analyzed using MASCOT DAEMON software version 2.2.2 (Matrix Science) with the SwissProt database (release SwissProt_2016). Searches for peptides were performed using the following parameters: full-trypsin specificity, a mass tolerance of 5 ppm on the parent ion and 0.5 Da on the MS/MS, carbamidomethylCys as static modification and oxidized Met as dynamic modification, and maximum number of missed cleavages set at 2. All peptide matches with a peptide score below a p-value of 0.05 were filtered. A protein was considered to be validated when at least two different peptides were detected in the same experiment. The false-positive rate for protein identification was estimated using the appropriate decoy database as below 1%. The number of MS/MS spectra per protein (spectral counts, SC) was determined for each sample and compared using the TFold method of the PatternLab software v3.2.0.3 as previously described, with a minimal SC cut-off set at 5 [29]. The relative quantitative proteomic analysis was calculated according to the method of normalized spectral abundance factors (NSAF) [18, 30]. The normalized spectral abundance factor is a ratio calculated by normalizing spectral counts (SC) obtained for a protein (i) by the respective molecular weight (MW) as follows:

$$NSAFi\;(\%)=\left(\frac{\frac{SCi}{MWi}}{\sum_{i=1}^{n}\left(\frac{SCi}{MWi}\right)}\right)\times 100$$

### 6. Cell culture

The HepG2 human hepatic cell line (HB-8065™) was obtained from the American Type Culture Collection (ATCC, Manassas, VA, USA). Cells were cultured in RPMI 1640 (Gibco™, Thermo Fisher Scientific) supplemented with 10% Hyclone™ FBS (ThermoFisher Scientific), and penicillin/streptomycin (100 U/mL and 100 μg/mL, respectively). Cells were incubated in a humidified incubator at 37°C and 5% CO_2_. Cells were passaged twice a week, keeping the confluence below 80%. Cells were used between passages 20 to 40.

### 7. Real-time cell impedance measurements

Real-time cell impedance measurements were performed with the xCELLigence system (ACEA Biosciences, San Diego, CA, USA). The background resistance of the E-plates (ACEA Biosciences) was determined using 100 μL culture medium. HepG2 cells were seeded in E-plates at 1.10^4^ cells per well. The E-plates were placed into the Real-Time Cell Analyzer (RTCA) station (ACEA Biosciences) and incubated at 37°C. Cells were grown for 24 h, with impedance recorded every minute for 12 h for the adhesion phase, then every 15 min for 36 h for the growth phase. After this, cells were exposed (n = 3) to pristine M-MSNs, M-MSNs-FBS corona or M-MSNs-HS corona, at 25, 50, and 100 μg/mL for 115 h, with monitoring every minute for the first 24 h (early effects) and every 15 minutes for the next 91 h (late effects). The impedance of control cells in the absence of M-MSNs was also recorded. Cell index (CI) raw data values were calculated using the RTCA software 2.0. Normalized cell indexes were calculated using this software at a selected normalization timepoint set just before the addition of nanoparticles. For a better understanding, the time-point zero of the X-axis on the figures corresponds to the NP exposure point. As a control, all nanoparticles were tested in acellular conditions and no interference on impedance measurements was observed, in accordance with other studies of cell impedance with nanoparticles [31].

## Results

### 1. Characterization of synthesized M-MSNs

**Fig 1:**
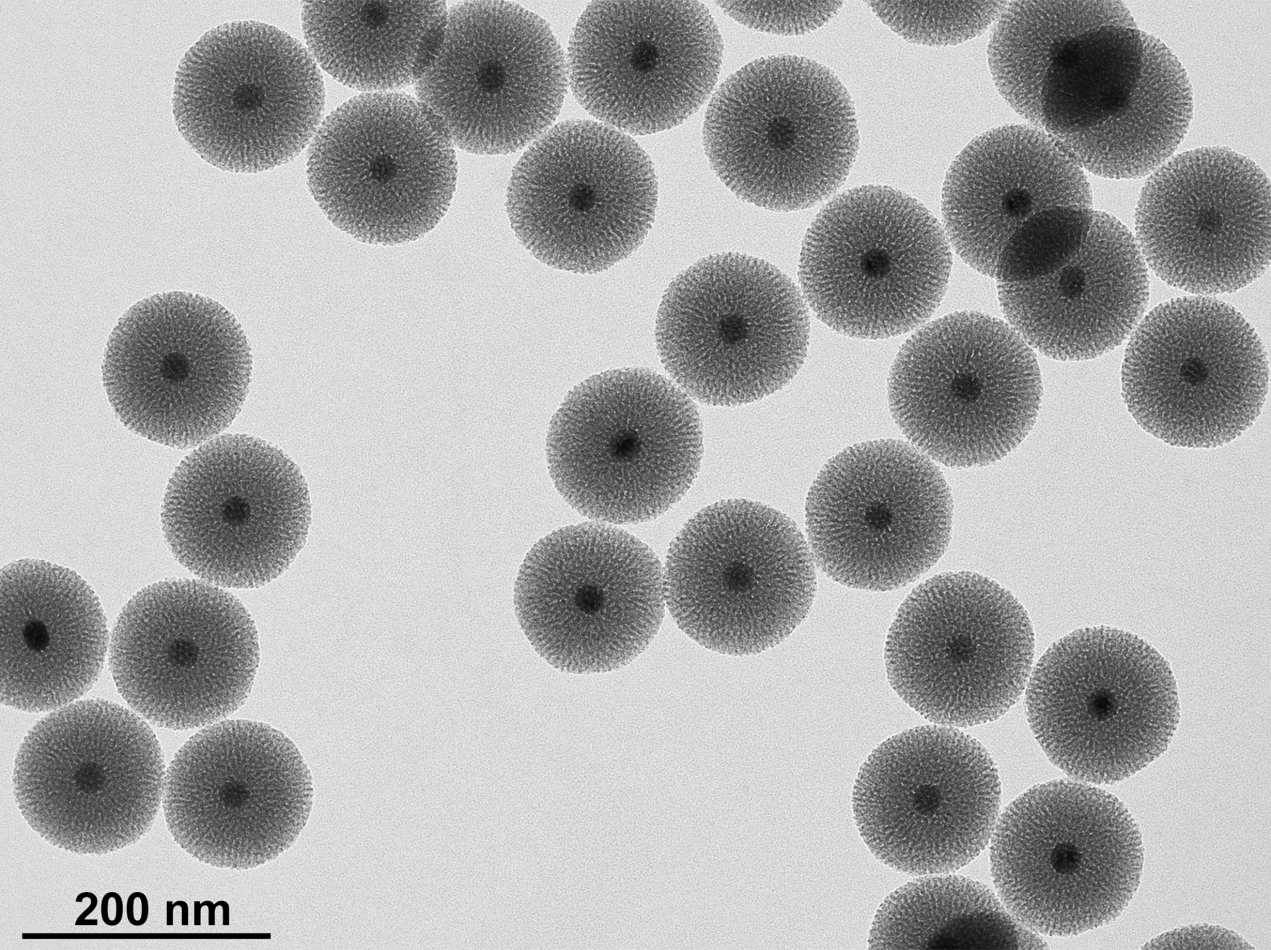
Transmission electron microscopy of synthesized magnetic mesoporous silica nanoparticles (M-MSNs). Mean diameter ± SD: 116.6 ± 2.1 nm.

M-MSNs were composed of an Fe_3_O_4_ nanocrystal core surrounded by a mesoporous silica shell [26]. In the pristine state, these NPs had a diameter of 116.6 nm (± 2.1) measured by TEM (Fig 1), and a hydrodynamic diameter of 143.5 nm (± 1.5) measured by DLS (Table 1). They were monodispersed and stable at physiological pH with a zeta potential of -39.1 mV (± 1.5).

**Table 1:** Hydrodynamic diameter and zeta potential measurements of M-MSNs, either pristine or in the presence of a preformed HS or FBS protein corona. Measurements were performed in physiological pH. Values are mean ± standard deviation (n = 3).

	Hydrodynamic Diameter (nm)	Polydispersity Index	Zeta Potential (mV)
**Pristine M-MSNs**	143.5 ± 1.5	0.111 ± 0.024	-39.1 ± 1.5
**M-MSNs—preformed FBS corona**	193.3 ± 6.5	0.187 ± 0.003	-20.1 ± 2.7
**M-MSNs—preformed HS corona**	220.8 ± 2.8	0.288 ± 0.018	-19.9 ± 1.7

After incubation of the M-MSNs in serum for 24 h, the zeta potential increased to -20.1 mV and -19.9 mV for FBS and HS, respectively. The hydrodynamic diameter increased to 193.3 nm and 220.8 nm for FBS and HS, respectively.

### 2. Identification of corona proteins adsorbed around nanoparticles

Proteomic analyses were performed to identify proteins contained within the corona formed around M-MSNs after 24 h of contact.

For FBS coronas more than 9,200 MS/MS spectra were analyzed, corresponding to 127 proteins (S1 Table). Proteins in the FBS corona included components of the complement system, apolipoproteins, and coagulation factors. The top 20 most abundant proteins in the corona (Table 2), included transporters (serum albumin, vitamin D-binding protein, fetal hemoglobin) and acute-phase response proteins (alpha-2-HS-glycoprotein, inter-alpha-trypsin inhibitor).

**Table 2:** Top 20 most abundant proteins in the fetal bovine serum corona. M-MSN samples exposed to sera were separated magnetically, washed with 1X PBS, and analyzed by nano-LC mass spectrometry as indicated in the Methods section. The number of MS/MS spectra per protein was determined for each sample and the relative quantitative proteomic analysis was calculated according to the method of normalized spectral abundance factors (NSAF). The twenty most abundant proteins are listed below.

Fetal Bovine Serum Protein Description	Uniprot accession	MS/MS Spectra	MW (Da)	NSAF (%)
Serum albumin	P02769	2296	69248	18.8
Alpha-2-HS-glycoprotein	P12763	1082	38394	16.0
Hemoglobin fetal subunit beta	P02081	176	15849	6.3
Apolipoprotein A-1	P15497	230	30258	4.3
Alpha-1-antiproteinase	P34955	282	46075	3.5
Serotransferrin	Q29443	373	77703	2.7
Alpha-fetoprotein	Q3SZ57	307	68543	2.5
Vitamin D-binding protein	Q3MHN5	196	53307	2.1
Inter-alpha-trypsin inhibitor heavy chain H4	Q3T052	363	101449	2.0
Fetuin-B	Q58D62	143	42636	1.9
Plasma serine protease inhibitor	Q9N2I2	147	45268	1.8
Apolipoprotein A-II	P81644	33	11195	1.7
Beta-2-glycoprotein 1	P17690	106	38227	1.6
Pigment epithelium-derived factor	Q95121	116	46200	1.4
Hemoglobin subunit alpha	P01966	38	15175	1.4
Gelsolin	Q3SX14	202	80681	1.4
Cystatin-C	P01035	40	16254	1.4
Complement C3	Q2UVX4	434	187135	1.3
Tetranectin	Q2KIS7	47	22130	1.2
Antithrombin-III	P41361	101	52314	1.1

For HS coronas, more than 13,600 MS/MS spectra were analyzed corresponding to 150 proteins (S2 Table). Proteins contained in the HS corona included components of the complement system, coagulation factors, and apolipoproteins. The top 20 most abundant proteins included complement factors (C3, B) and some IgGs (Table 3).

**Table 3:** Top 20 most abundant proteins in the human serum corona. M-MSN samples exposed to sera were separated magnetically, washed extensively with 1X PBS, and analyzed by nano-LC mass spectrometry as indicated in the Methods section. The number of MS/MS spectra per protein was determined for each sample and the relative quantitative proteomic analysis was calculated according to the method of normalized spectral abundance factors (NSAF). The twenty most abundant proteins are listed below.

Human Serum Protein Description	Uniprot accession	MS/MS Spectra	MW (Da)	NSAF (%)
Serum albumin	P02768	4268	69321	25.5
Apolipoprotein A-I	P02647	324	30759	4.4
Ig kappa chain C region	P01834	120	11602	4.3
Ig gamma-1 chain C region	P01857	348	36083	4.0
Ig lambda-2 chain C regions	P0CG05	73	11287	2.7
Alpha-2-HS-glycoprotein	P02765	254	39300	2.7
Complement C3	P01024	1167	187030	2.6
Serotransferrin	P02787	398	77014	2.1
Hemopexin	P02790	253	51643	2.0
Beta-2-glycoprotein 1 (Apolipoprotein H)	P02749	157	38273	1.7
Vitamin D-binding protein	P02774	208	52929	1.6
Alpha-1-antitrypsin	P01009	183	46707	1.6
Apolipoprotein A-IV	P06727	153	45371	1.4
Apolipoprotein A-II	P02652	36	11168	1.3
Ig gamma-3 chain C region	P01860	121	41260	1.2
Plasminogen	P00747	254	90510	1.2
Apolipoprotein E	P02649	97	36132	1.1
Complement factor B	P00751	206	85479	1.0
Gelsolin	P06396	187	85644	0.9
Apolipoprotein D	P05090	46	21262	0.9

The two coronas were similar, with nine homologous proteins in the top 20, including serum albumin (P02769, P03768), complement component C3 (Q2UVX4, P01024), serotransferrin (Q29443, P02787), apolipoprotein A-1 (P15497, P02647), gelsolin (Q3SX14, P06396), alpha-2-HS-glycoprotein (P12763, P02765), vitamin D-binding protein (Q3MHN5, P02774), and beta-2-glycoprotein 1 (P17690, P02749).

**Fig 2:**
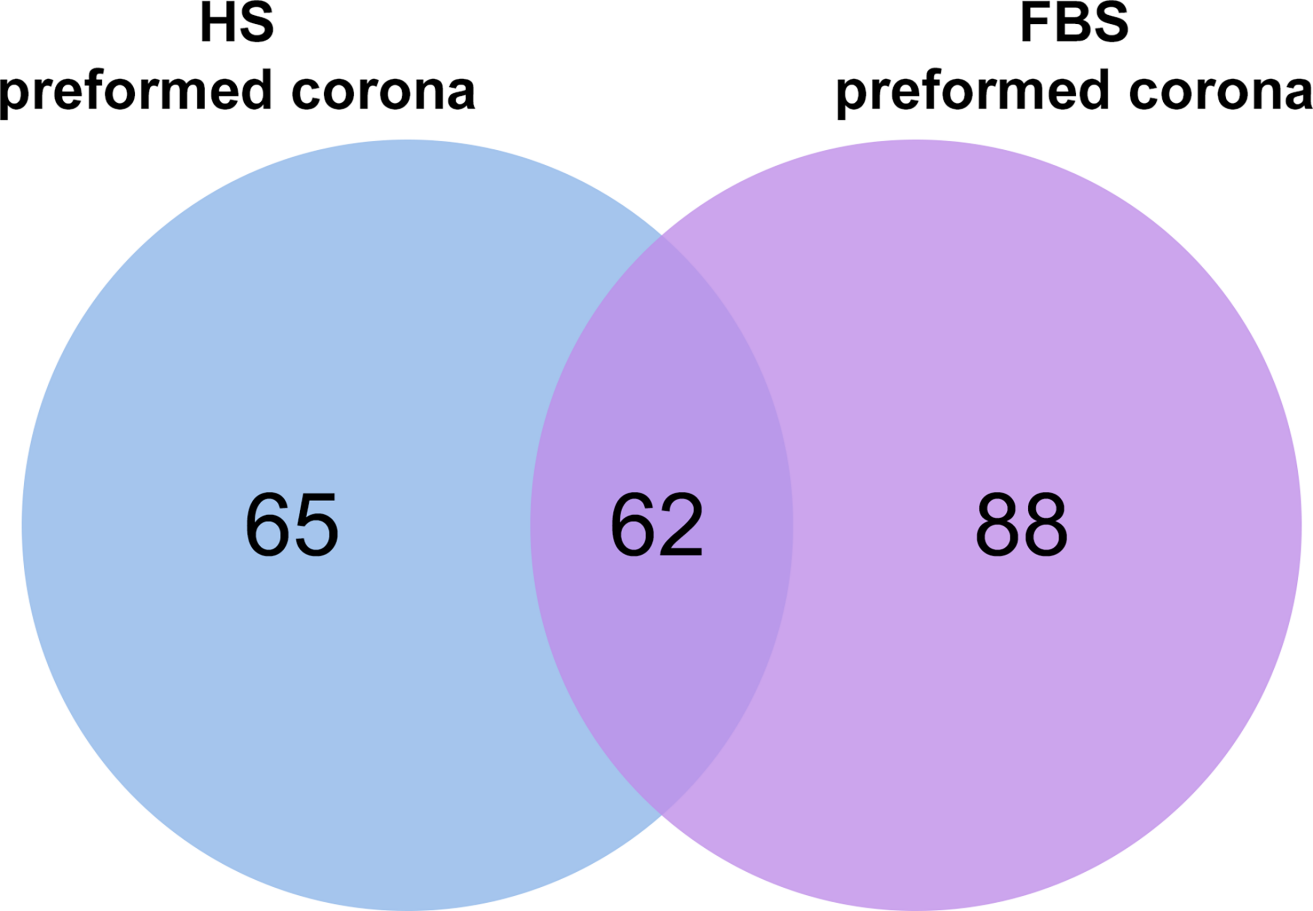
VENN diagram. This diagram represents the number of specific proteins per preformed corona identified by nano-LC MS/MS. The two coronas present 62 homologous proteins.

VENN diagram (Fig 2) showed homologous proteins between preformed coronas. These proteins represented 49% of the FBS preformed corona and 41% of the HS preformed corona. However, despite the large part of homologies between both coronas, these homologous proteins had quantitative differences, as represented by their normalized spectral abundance factors (NSAF) in Tables 2 and 3. For instance, Serum Albumin (FBS: P02769; HS: P02768), represented 18.8 and 25.5% of the FBS and HS preformed coronas, respectively, or Alpha-2-HS-Glycoprotein (FBS: P12763; HS: P02765), constituted 16.0% of the FBS versus 2.7% of the HS preformed corona.

**Fig 3:**
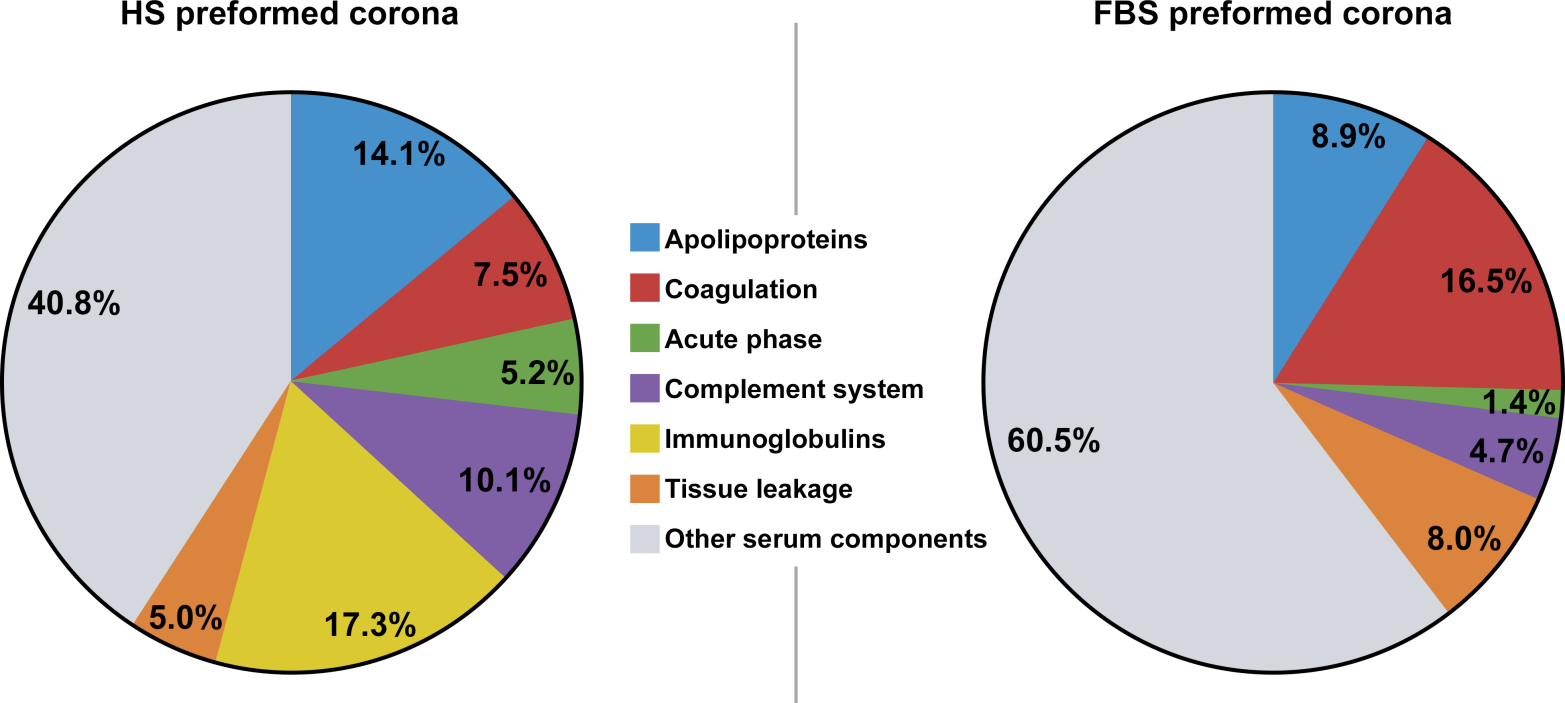
Classification of corona components. Proteins identified in respective preformed coronas by nano-LC MS/MS were grouped according to their biological processes in the blood system (Uniprot DB). Values are expressed as percentages.

Proteins identified in both preformed coronas were classified according to their functional annotations from the Uniprot database (Fig 3). We observed firstly the complete absence of Immunoglobulins (Ig) in the FBS corona, while these Ig constituted 17.3% of the HS preformed corona. Apolipoproteins were more frequently present in the HS (14.1%) than FBS (8.9%) corona, while Coagulation components constituted 7.5% of the HS corona and 16.5% of the FBS corona.

### 3. Cytotoxicity of nanoparticles recorded by real-time cell impedance

#### 3.1 HepG2 cells exposed to pristine M-MSNs

**Fig 4:**
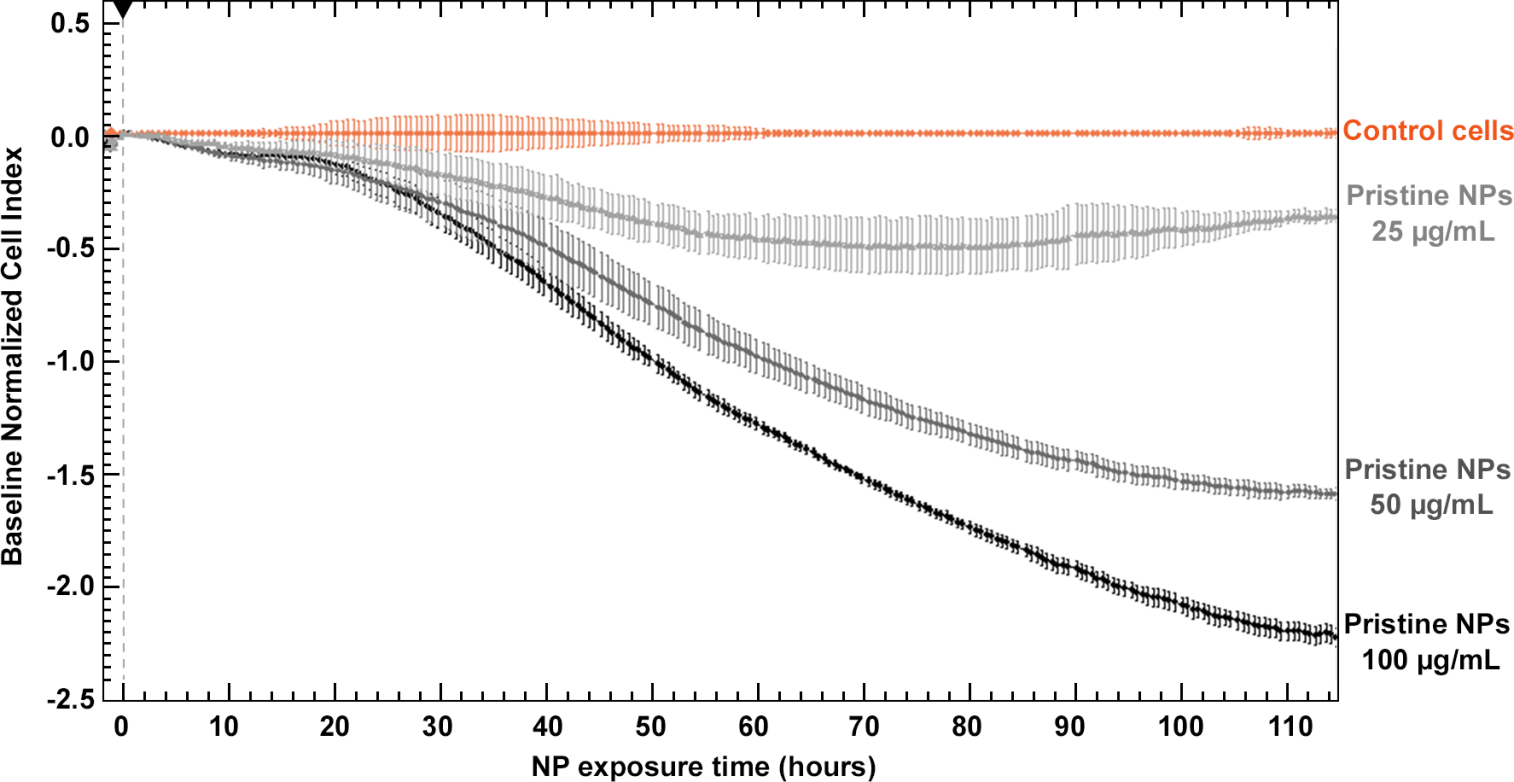
Real-time cell index (CI) monitoring of HepG2 cells (n = 3) exposed to 25, 50, or 100 μg/mL pristine M-MSNs for 115 hours. All curves represent the mean of three curves with recorded standard deviations at each time-point. The black arrow represents the starting point of exposure. Cell index was normalized to baseline (control cells) to ensure inter-dose comparison.

Real-time cell impedance monitoring was used to investigate the effects of pristine M-MSNs on human HepG2 hepatocytes, including viability, adhesion, and morphology. Fig 4 represents a multiple-dose test of the exposure of cells to pristine M-MSNs.

Pristine M-MSNs induced a dose-dependent reduction in the cell index (CI), as compared with control cells. At 50 and 100 μg/mL, these nanoparticles induced a drastic effect in a biphasic pattern, with a slow decrease in the CI over the first 20 h of exposure followed by a strong decrease to the end of the experiment. At 25 μg/mL, we observed a biphasic effect composed of a slow decrease in the CI during the first 60 h of exposure followed by a slow increase to the end of the experiment. At the end of the experiment, i.e. after 115 h exposure, the dose-dependent trend was conserved. At 25 and 50 μg/mL, the toxicity of M-MSNs on HepG2 cells was 7- and 1.5-fold lower than at 100 μg/mL, respectively.

#### 3.2 HepG2 cells exposed to M-MSNs in the pristine state or covered with serum proteins

**Fig 5:**
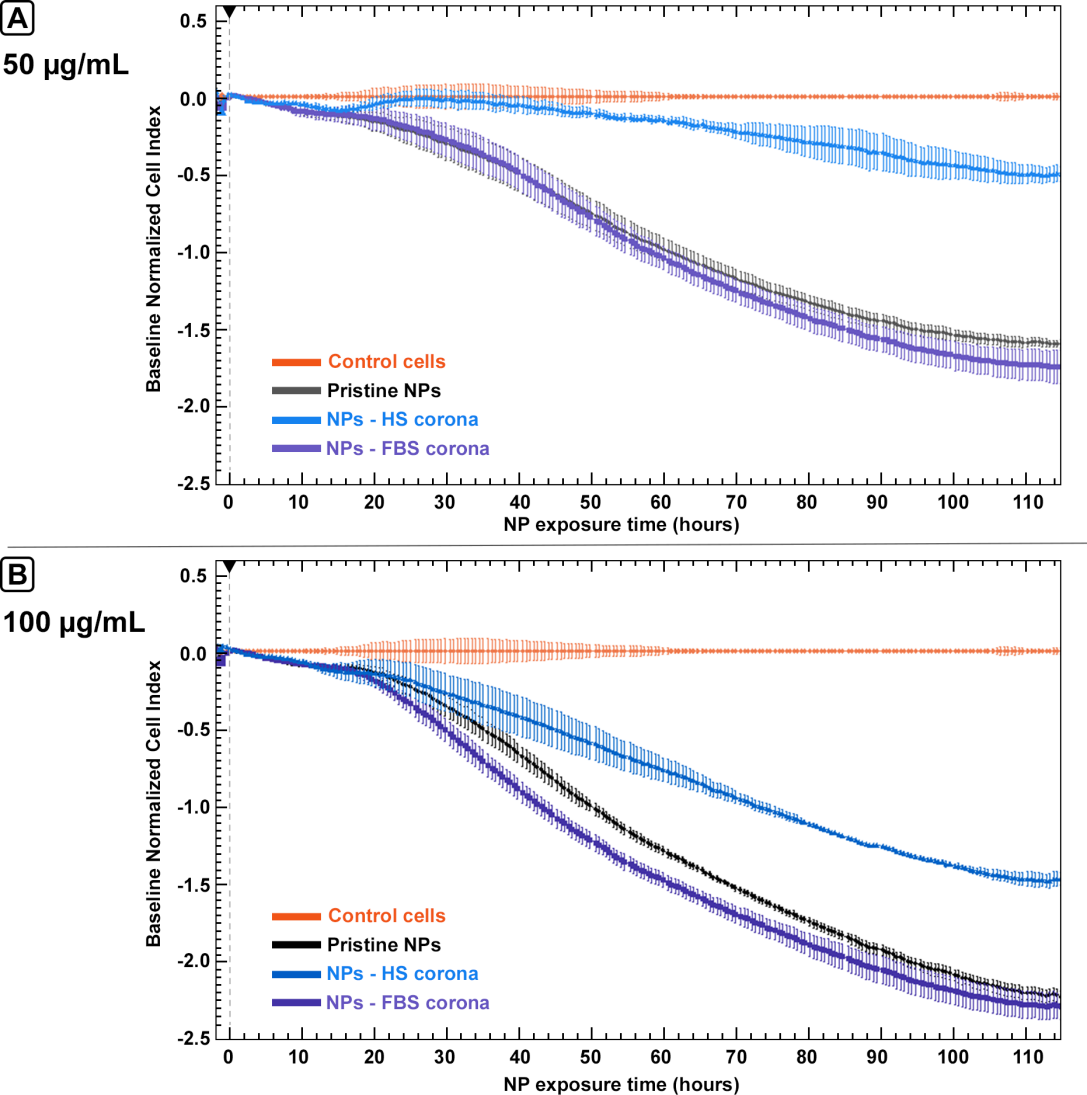
Real-time cell index (CI) monitoring of HepG2 cells (n = 3) exposed to A) 50 μg/mL, B) 100 μg/mL M-MSNs, in a pristine state or covered with serum proteins, for 115 hours. All curves represent the mean of three curves with recorded standard deviations at each time-point. The black arrow represents the starting point of exposure. Cell index was normalized to baseline (control cells).

HepG2 cells were exposed to pristine M-MSNs or protein-covered M-MSNs. Fig 5 shows that M-MSNs covered with bovine proteins behaved as pristine M-MSNs, i.e. showing a biphasic pattern with a slow decrease in the CI for the first 20 h followed by a rapid decrease until the end of the experiment. This effect was visible at the two concentrations tested (50 and 100 μg/mL). Interestingly, M-MSNs covered with human proteins induced a quite different effect, with a less disturbed CI, likely reflecting much lower cytotoxicity than with pristine and bovine protein-covered M-MSNs. At the end of the experiment using HS instead of FBS decreased toxicity by 4-fold and 1.5-fold at 50 and 100 μg/mL M-MSNs, respectively.

## Discussion

**Fig 6:**
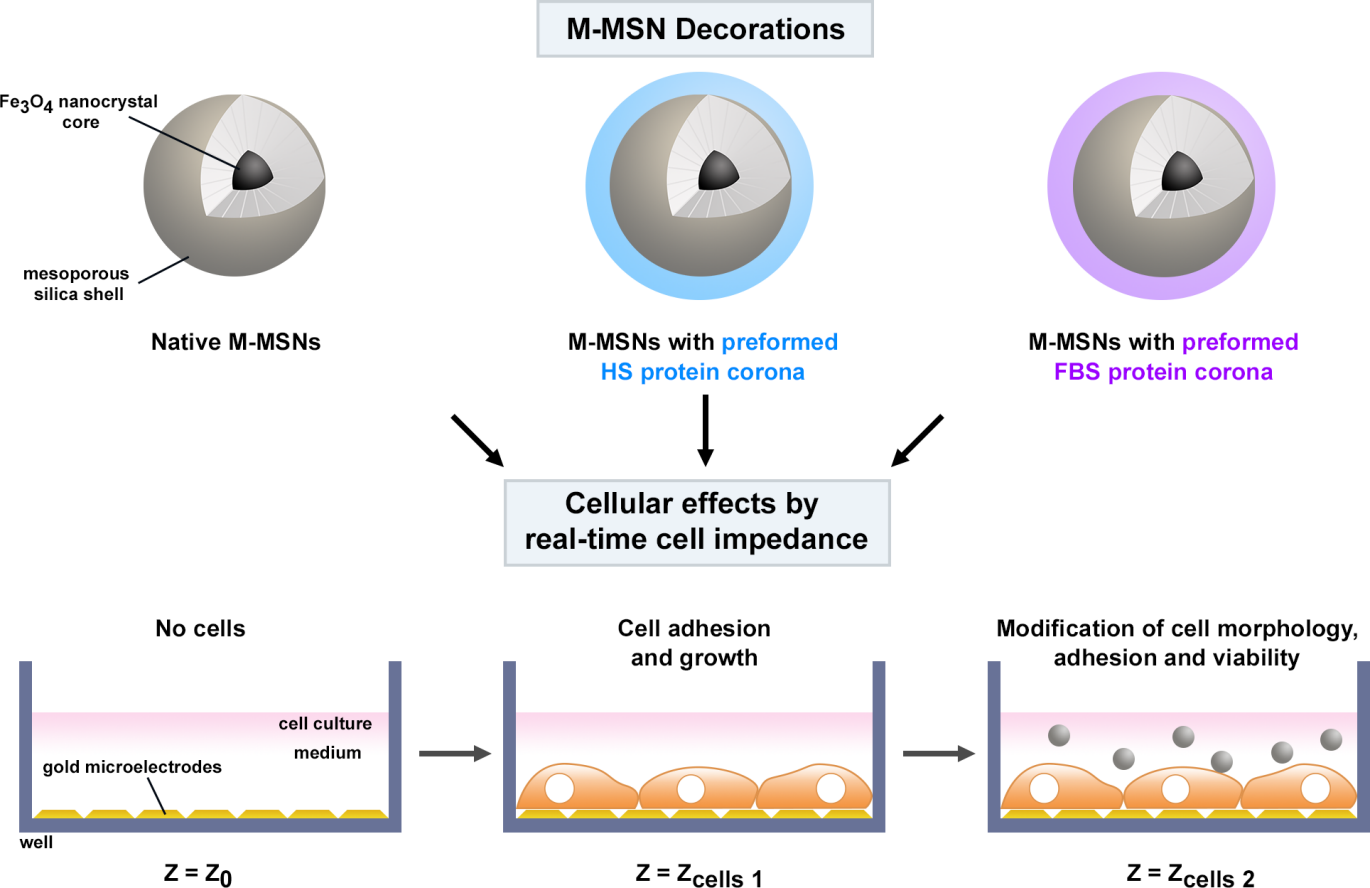
Strategy used to investigate the impact of the corona on toxicological outcomes using real-time cell impedance.

Magnetic mesoporous silica nanoparticles (M-MSNs) are nanoparticles with a great potential in theranostics. Nevertheless, their behavior in biological fluids needs further investigation. In particular, the formation of a corona of proteins around the nanoparticles influences nanoparticle toxicity by triggering cellular mechanisms that may be totally different from those associated with pristine nanoparticles [14, 25, 32–36]. The main objective of this study was to investigate whether the species origin of the serum proteins forming the corona around the M-MSN influences the nanoparticle toxicity in cell cultures *in vitro*. The strategy used for this investigation was to preform coronas around M-MSNs before the exposure of cells, by incubating the M-MSNs in either fetal bovine serum (FBS) or human serum (HS) (Fig 6). We then monitored the impact of these protein-coated particles on HepG2 cells using real-time cell impedance technology. Cell-based phenotypic assays are being more often used in drug discovery, and thus label-free technologies based on impedance measurements represent a powerful tool. Atienzar *et al*. demonstrated a very good correlation between cell index (CI) determined by RTCA and cell viability measured by a standard and traditional assay in HepG2 cells exposed to a set of 50 compounds [27]. The CI reflects modifications of both cell morphology and cell viability.

M-MSNs were synthesized and characterized by TEM and DLS, showing homogeneous spheres (Fig 1) that were stable at physiological pH (Table 1) as described previously [26]. With preformed FBS or HS coronas, nanoparticle size increased. The proteins enabled the nanoparticles to remain in a dispersed form, as indicated by their low polydispersity indexes (Table 1).

In the acellular condition, the proteins adsorbed around M-MSNs after 24 h were identified by mass spectrometry and the results were consistent with those reported in the literature [19, 36–39]. A panel of proteins was identified, including abundant serum proteins such as albumin, and well-known protein families including components of the complement system, coagulation factors, and apolipoproteins. Very recently Strojan *et al*, 2017 described a list of 30 proteins found in the FBS-corona of silica NPs; all these proteins are included in our data set (S1 Table), especially complement factor H, complement C3 and complement C4, all part of the complex complement system [39]. The components of both coronas included 62 homologous proteins as depicted in Fig 2. These proteins represented 49% of the FBS preformed corona and 41% of the HS preformed corona. However, despite the large homology between both coronas, these proteins showed quantitative differences, as represented by their normalized spectral abundance factors (NSAF) in Tables 2 and 3. For instance, Serum Albumin (FBS: P02769; HS: P02768), represented 18.8 and 25.5% of the FBS and HS preformed coronas, respectively. Alpha-2-HS-glycoprotein (P12763) constitutes 16.0% of the bovine corona whereas the homologous human protein (P02765) represents only 2.7% of the human corona. We observed the complete absence of Immunoglobulins (Ig) in the FBS corona while these Ig constituted 17.3% of the HS preformed corona (Fig 3). This result is expected since fetal serum is not equipped with antibodies. Apolipoproteins were more frequently present in HS corona (14.1%) than in FBS corona (8.9%), while coagulation components constituted 7.5% of the HS corona and 16.5% of the FBS corona (Fig 3). This difference could be a reason for the increased toxicity of bovine vs. human serum.

In an *in vitro* toxicity assay, the cell culture medium contains protein serum and a similar corona formation is expected. Some studies have described the effect of serum on nanomaterial toxicity [14, 19, 25, 35, 40–42]. Corona formation was found to affect hemolysis, thrombocyte activation, nanoparticle uptake and endothelial cell death [19]. When in contact with a biological environment, the protein corona confers on NPs a new bioidentity. Some of the proteins in this corona can be recognized by specific cell membrane receptors, triggering internalization mechanisms that are different to those generated by pristine NPs [40]. Once inside cells, NPs may cause adverse effects and permanent cell damage. Mechanisms including oxidative stress, inflammation, genetic instability, and the inhibition of correct cell division may be involved and may contribute to cell death [5].

In this context, hepatic HepG2 cells were exposed to three different concentrations of pristine M-MSNs. A dose-dependent response of the HepG2 cell index was observed with exposure to pristine nanoparticles (Fig 4). HepG2 cells were then exposed to M-MSNs covered with coronas of two different species origins, each at two different concentrations (Fig 5). M-MSNs covered with bovine proteins induced similar effects to pristine NPs at both doses tested 50 μg/mL (Fig 5A) and 100 μg/mL (Fig 5B). This result is related to the kinetics of corona formation [18, 23, 36]. Indeed, corona formation begins as soon as NPs are in contact with the proteins contained in the culture medium. We have previously shown that after 30 seconds the corona has already formed around the NP [18]. Thus, by the time the nanoparticles and cells come into contact, the corona has already formed around the pristine M-MSNs, producing similar effects to those elicited by a 24-hour preformed FBS corona. The formation of the corona occurs within the first minutes.[18, 19]. The fact that the cellular effects are similar between the 24h- and the minute-corona proves that the subsequent exchanges between the corona proteins and the surrounding medium are of little importance on the cellular toxicity of NP. Nevertheless, Fig 5 also clearly shows that M-MSNs with a preformed human corona induced much lower toxicity on HepG2 cells than M-MSNs with a preformed bovine corona. Using HS instead of FBS mitigated M-MSN toxicity by 4-fold and 1.5-fold at 50 and 100 μg/mL, respectively.

**Fig 7:**
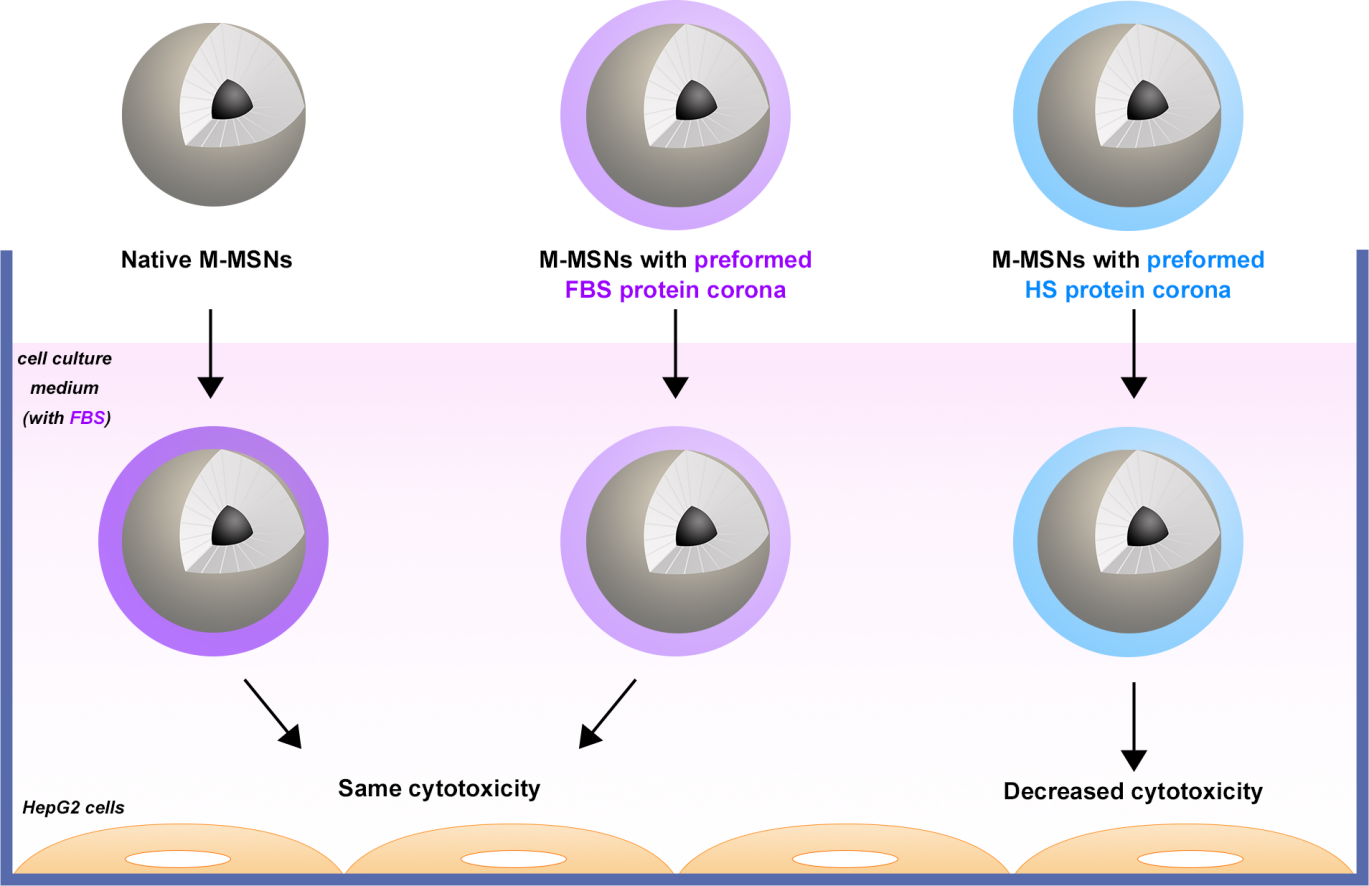
M-MSNs with a protein corona preformed in fetal bovine serum induced the same effects as pristine M-MSNs in HepG2 cells, because the corona forms instantly around the pristine M-MSNs when they encounter the cell medium containing 10% FBS. Using HS instead of FBS to preform coronas mitigated M-MSN (50 μg/mL) toxicity by 4-fold in HepG2 cells.

Consequently our findings show that sera from different species induce different cellular behaviors. This discrepancy may be explained by the presence of cell membrane receptors able to distinguish between human proteins and homologous proteins of other species [24]. The latter are likely to be preferentially internalized to be destroyed in lysosomes [43]. When protected by human proteins, M-MSNs may appear more “neutral” to the human cells, as summarized in Fig 7. It is likely that some “markers of self” are present in sera. For example, CD47 is described as a “marker of self” [44] and is present at the surface of exosomes in normal human serum [45]. The phagocytes possess a cell surface receptor similar to immunoglobulin for CD47 (SIRPα) which binds exclusively to human but not bovine CD47 [46]. CD47 or another circulating human “marker of self” protein effectively acting as a cellular passport might be recognized at the HepG2 cell surface by an as-yet unknown receptor. This could explain the species recognition that we observed.

For economic reasons, most *in vitro* toxicity assays use FBS in the cell culture media. Taking into consideration the results of this study, it is then possible that the toxicities of nanoparticles might sometimes be overestimated when FBS is used in this type of assay. For *in vitro* assays of nanoparticles intended for drug delivery, the use of nanoparticles preincubated in solutions close to the biological composition of human blood would be more representative of an *in vivo* situation and would enable their toxicity to human cells to be determined more accurately. From a broader perspective, the route of exposure should be taken into account in developing a preincubation solution for nanoparticles, in order to represent the *in vivo* scenario as closely as possible. For economic reasons, it is probably not currently possible to replace the use of FBS with HS in cell cultures for high-throughput *in vitro* toxicity screening. Some solutions that have been proposed include alternative culture media to avoid the use of FBS in cell culture [47]. Alternative media (human platelet lysates for instance) to maintain the cell culture growth necessitate a case-by-case basis optimization. The toxicity of nanoparticles in such a xeno-free medium would eliminate the side effect of bovine serum. However, this introduces questions of relevance and biological equivalence, because *in vivo* exposures do not occur in the absence of host proteins [48]. Another solution is to induce a human corona before cell culture assays by preincubating nanoparticles in a human protein solution, chosen according to the route of exposure (injection, inhalation, etc.). In the case of injectable nanocarriers, the use of human serum before testing the toxicity of NP would be more relevant, in order to more closely represents the *in vivo* situation. This solution could limit the influence of the biological medium (containing FBS or alternative mixtures) used in these *in vitro* assays. Other developments will be needed, such as spheroid 3D models, which have been demonstrated to be less sensitive to the toxic effects of NPs as compared with 2D cultures of the same cells (HepG2) [49].

## Conclusions

Magnetic mesoporous silica nanoparticles (M-MSNs) are being developed for use as injectable drugs, but their safety must first be proven. Nanotoxicological testing is firstly assessed using *in vitro* methods established for the hazard characterization of chemicals. Nevertheless, special considerations are needed to assess *in vitro* effects of nanoparticles, compared to molecular forms of drugs. Unlike chemicals, the formation of a protein corona around nanoparticles complicates these tests, in particular because of the presence of fetal bovine serum as a common additive in standard culture media of human cells. In this study we showed that M-MSNs surrounded by a corona originating from fetal bovine serum in the cell medium induced toxic effects on HepG2 cells that were greater than with M-MSNs carrying a preformed human serum corona. This situation may lead to the overestimation of nanoparticle toxicity using standardized protocols. It will be necessary to minimize the impact of the corona on toxicity assays. The route of nanocarrier delivery must be considered for hazard testing, and preforming a corona with human serum seems to be more appropriate for *in vitro* testing than using nonhuman serum. To provide solid and reliable results and to offer a robust alternative to animal testing, *in vitro* cytotoxicity assays must closely represent conditions *in vivo*.

## Supporting information

S1 Table List of protein relative abundances in fetal bovine corona, obtained by NSAF ratio. (XLSX)

S2 Table List of protein relative abundances in human corona, obtained by NSAF ratio. (XLSX)
